# Decitabine-Induced Changes in Human Myelodysplastic Syndrome Cell Line SKM-1 Are Mediated by FOXO3A Activation

**DOI:** 10.1155/2017/4302320

**Published:** 2017-10-16

**Authors:** Wen Zeng, Hanjun Dai, Ming Yan, Xiaojun Cai, Hong Luo, Min Ke, Zeming Liu

**Affiliations:** ^1^Department of Ophthalmology, Zhongnan Hospital of Wuhan University, Wuhan, China; ^2^Department of Breast and Thyroid Surgery, Union Hospital, Tongji Medical College, Huazhong University of Science and Technology, Wuhan 430022, China

## Abstract

The epigenetic silencing of tumor suppressor genes in myelodysplastic syndromes (MDS) can potentially confer a growth advantage to individual cellular clones. Currently, the recommended treatment for patients with high-risk MDS is the methylation agent decitabine (DAC), a drug that can induce the reexpression of silenced tumor suppressor genes. We investigated the effects of DAC treatment on the myeloid MDS cell line SKM-1 and investigated the role of FOXO3A, a potentially tumor-suppressive transcription factor, by silencing its expression prior to DAC treatment. We found that FOXO3A exists in an inactive, hyperphosphorylated form in SKM-1 cells, but that DAC both induces FOXO3A expression and reactivates the protein by reducing its phosphorylation level. Furthermore, we show that this FOXO3A activation is responsible for the DAC-induced differentiation of SKM-1 cells into monocytes, as well as for SKM-1 cell cycle arrest, apoptosis, and autophagy. Collectively, these results suggest that FOXO3A reactivation may contribute to the therapeutic effects of DAC in MDS.

## 1. Introduction

FOXO3A, also known as forkhead in rhabdomyosarcoma-like protein 1 (FKHRL1), is a transcription factor with important roles in embryonic development, differentiation, and tumorigenesis [1]. It is characterized by the presence of the distinctive forkhead DNA binding domain, a highly conserved winged helix motif, and regulates the transcription of genes involved in a variety of processes, including cell cycle regulation [2, 3], apoptosis [4, 5], DNA repair [6], and autophagy [7–9]. FOXO3A function is regulated by posttranslational modifications such as phosphorylation, acetylation, and ubiquitination, which ultimately affect its nuclear/cytoplasmic transport and hence cellular location [10–12]. FOXO3A is considered to be a potential tumor suppressor gene and is involved in the regulation of differentiation in various cell types [13–16]. Furthermore, FOXO3A is inactivated, and its target genes are downregulated, following phosphorylation by oncogenic kinases such as AKT, MAPK1, and IKK, which are upregulated in many tumors [17–19]. Interestingly, the reexpression and activation of FOXO3A in tumor cells reportedly have potential in antitumor treatment [20].

A previous study showed that the epigenetic silencing of tumor suppressor genes could confer a growth advantage to a subgroup of myelodysplastic syndrome (MDS) cell clones. Such epigenetic modifications are reversible, and the silenced genes can be reactivated using methyltransferase inhibitors such as decitabine (DAC). Indeed, high doses of DAC are known to impair gene methylation, resulting in the activation of various cellular processes such as apoptosis [21]. On the other hand, at low doses, DAC is incorporated into newly synthesized double-stranded DNA during the S phase of the cell cycle without affecting elongation and induces cell cycle arrest and cellular differentiation [22, 23]. Such S phase-specific DAC incorporation may be responsible for a plateau in DAC activity that was observed in AML cell lines, wherein cellular activity could not be lowered beyond 40% even when the DAC concentration was increased to 50 *μ*M [24]. However, it is possible to enhance the impact of DAC on cellular activity by extending the treatment duration; a reduction to just 15% of the original activity has been observed after 6 days of DAC treatment [24]. Other reports have confirmed that DAC-induced inhibition of cellular proliferation depends mainly on treatment duration. Additionally, DAC-induced differentiation has been primarily observed at low doses, while high doses tend to be cytotoxic [25]. The optimal dosage of DAC has been explored; Cmax values of 0.3–1.6 *μ*M have been reported in human plasma [26–28], while the reported EC_50_ for a variety of AML cell lines reportedly ranged from 0.4 to 0.8 *μ*M [24, 29].

In this study, we investigated the effects of DAC on differentiation, apoptosis, cell cycle arrest, and autophagy using the myeloid MDS tumor cell line SKM-1. Our model used a low concentration of DAC (0.5 *μ*M) and a duration of treatment of 6 days. Furthermore, we investigated the role of FOXO3A in DAC-dependent processes by measuring the expression levels and activity of this gene and its downstream targets following DAC treatment.

## 2. Materials and Methods

### 2.1. Cell Culture and DAC Treatment

Human myelodysplastic syndrome cell line SKM-1 was a gift from Professor Li Chunrui at the Department of Hematology, Tongji Medical College, Tongji Hospital, China. SKM-1 cells were cultured in Dulbecco's modified eagle medium (DMEM; Gibco Life Technologies, Grand Island, NY, USA) containing 10% FBS (Gibco Life Technologies) and 1% penicillin-streptomycin at 37°C in 5% CO_2_. When cells reached the logarithmic growth phase, they were seeded at a density of 5 × 10^5^ cells/well in 6-well plates and treated with 0.5 *μ*M DAC (Xian-Janssen Pharmaceutical, Xian, China) for 6 days. Every 48 hours, the medium in the wells was replaced with fresh medium containing 0.5 *μ*M DAC.

### 2.2. Cellular Transfection

Cells were transfected with either *Silencer*® Select FOXO3a (cat. number 4392420) or *Silencer* Select Negative Control (cat. number 4390843) siRNAs, both of which were purchased from Ambion (Thermo Fisher Scientific, Waltham, MA, USA), and Lipofectamine® 3000 reagent (Invitrogen, Carlsbad, CA, USA) according to the manufacturer's instructions. Briefly, SKM-1 cells were washed twice in phosphate-buffered saline (PBS) before being resuspended in Opti-MEM medium (Gibco Life Technologies) at a density of 1 × 10^6^ cells/ml. Five hundred microliters of this cell suspension was then diluted 2-fold in Opti-MEM medium and added to 6-well plates, giving 5 × 10^5^ cells/well. To prepare the siRNA liposomes, 3.75 *μ*l Lipofectamine 3000 reagent and 75 pg of the appropriate siRNA were each gently mixed with individual 125 *μ*l aliquots of Opti-MEM, before being combined, and incubated at room temperature for 5 minutes. All 250 *μ*l of the liposome mixture was then added to each well, and cells were incubated at 37°C in 5% CO_2_. After 24 hours of transfection, the medium was replaced with fresh DMEM. The transfected cells were either cultured or treated with 0.5 *μ*M DAC for 48 hours as required.

### 2.3. Assessment of Apoptosis Using Hoechst 33342 Staining

To examine the degree of apoptosis, DAC-treated cells were collected and washed once in PBS, before being counted and resuspended in PBS at a concentration of 1 × 10^7^ cells/ml. Cells were then dried naturally onto antioff slides (ZSGB-BIO, Beijing, China) at room temperature, before being fixed in 4% paraformaldehyde at room temperature for 15 minutes and washed three times for 5 minutes in PBS. One hundred microliters of Hoechst 33342 stain (Beyotime Institute of Biotechnology, Nanjing, Jiangsu, China) was added to each slide, and the slides were incubated for 5 minutes at room temperature in darkness within a humidity chamber. Finally, slides were washed three times for 5 minutes in PBS; then, within 1 hour, they were visualized and photographed using a fluorescence microscope (Olympus TH4-200).

### 2.4. Assessment of Apoptosis Using Flow Cytometry

Apoptosis assays were performed using an Annexin V-FITC/PI apoptosis kit (Beyotime Biotech) according to the manufacturer's instructions. Briefly, the DAC-treated cells were collected and washed three times in PBS before being counted and resuspended 2 × 10^5^ in 200 *μ*l binding buffer. Ten microliters of Annexin V-FITC and 5 *μ*l propidium iodide (PI; Beyotime Biotech) was then added to the cell suspensions, and samples were incubated in darkness for 15 minutes. Within 1 hour, apoptotic cells were detected by flow cytometry using a flow cytometer (FACSCanto II, BD Biosciences, Franklin Lakes, NJ, USA). Experiments were performed in triplicate.

### 2.5. Assessment of the Cell Cycle Using PI Staining

The cell cycle stages of the SKM-1 cells were assessed using a PI staining assay kit (Beyotime Biotech) according to the manufacturer's instructions. Briefly, collected cells were washed once in cold PBS and resuspended in 75% ethanol that had been precooled to −20°C. The cells were counted and their concentration was adjusted to 2 × 10^6^ cell/tube using 75% ethanol, and they were fixed at −20°C between 1 and 24 hours. Cells were then pelleted by centrifugation at 2000*g* for 5 minutes, washed twice with cold PBS, and resuspended in 200 *μ*l PBS. RNase was then added to each tube to a final concentration of 100 *μ*g/ml, mixed, and incubated at 37°C for 30 mins. Next, 235 *μ*l PBS and 60 *μ*l PI (50 *μ*g/ml final concentration) were added to each tube, mixed, and incubated in darkness at 37°C for 30 minutes. Cell cycle stages were examined by flow cytometry within an hour using a flow cytometer (FACSCanto II, BD Biosciences). Experiments were performed in triplicate.

### 2.6. Detection of Cell Surface Markers

To assess the expression of cell surface markers, DAC-treated cells were collected and washed three times in PBS before being counted and resuspended in 200 *μ*l PBS at a concentration of 1 × 10^6^ cells/tube. Next, either 10 *μ*l FITC-conjugated anti-CD14 or APC-conjugated anti-CD11b antibodies or FITC- or APC-conjugated isotype controls (Becton Dickinson) were added to the cells as appropriate, mixed gently, and incubated in darkness at room temperature for 15 minutes. Cells were then washed once in PBS and resuspended in 200 *μ*l PBS, and within 1 hour, the surface markers were analyzed by flow cytometry using a flow cytometer (FACSCanto II, BD Biosciences). Experiments were performed in triplicate.

### 2.7. Western Blot Analysis

Proteins were extracted from cells using RIPA buffer with 1× protease/phosphatase inhibitor cocktail (Cell Signaling Technology, Danvers, MA, USA) according to the manufacturer's instructions, and the concentration of total protein was determined using the BCA Protein Assay kit (Beyotime Biotech) according to the manufacturer's protocol. Thirty micrograms of total protein was separated using a sodium dodecyl sulfate polyacrylamide gel electrophoresis kit (Beyotime Biotech) according to the manufacturer's instructions, before being transferred onto polyvinylidene fluoride membranes (Millipore, Billerica, MA, USA) in transfer buffer (200 mM glycine, 40 mM Tris, and 20% methanol) at 240 mA for between 30 and 90 minutes, depending on the molecular weight of the proteins. Membranes were then blocked with 5% BSA (CST) in TBS with 0.1% TBST (CST) for 1 hour at 37°C before incubating with the described primary antibodies (Supplementary Table 1 available online at https://doi.org/10.1155/2017/4302320; diluted according to instructions) overnight at 4°C. Membranes were then washed three times for 5 minutes in 0.1% TBST and then incubated for 1 hour at 37°C with horseradish peroxidase-conjugated anti-rabbit secondary antibody (Cell Signaling Technology) at a dilution of 1 : 3000. Membranes were washed a further three times for 5 minutes in 0.1% TBST before ECL Chemiluminescent Substrate reagent (Cell Signaling Technology) was added and images were obtained using Bio-Rad ChemiDoc™ XRS+ Imaging System (Bio-Rad Laboratories, Hercules, CA, USA). The intensity of bands was quantified using Image Lab software version 2.0 (Bio-Rad Laboratories, Hercules, CA, USA), and GAPDH was used as an internal standard.

### 2.8. Statistical Analysis

Results were obtained from three independent replicate experiments and are expressed as mean ± standard deviation. Data were analyzed using SPSS version 13.0 software (SPSS Inc., Chicago, IL, USA). The significance of differences between groups was assessed using Student's *t*-test, and statistical significance was defined as *P* < 0.05.

## 3. Results

### 3.1. FOXO3A Contributes to DAC-Induced SKM-1 Cell Differentiation

**Figure 1:**
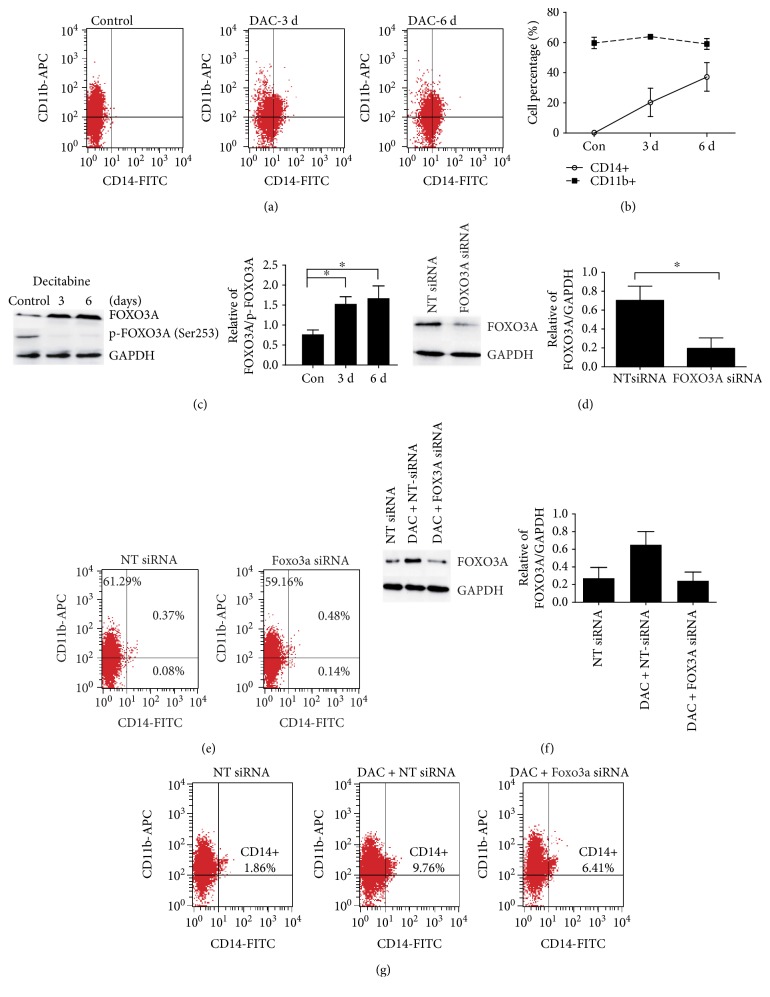
FOXO3A contributes to DAC-induced SKM-1 cell differentiation. (a, b) CD14 (marker for monocytes), but not CD11b (marker for myeloid cells), was significantly induced in SKM-1 cells treated with 0.5 *μ*M DAC for 3 and 6 days. (c) Western blot showed that DAC treatment increases FOXO3A expression and FOXO3A/p-FOXO3A ratio. (d) Western blot showed that siRNA targeting FOXO3A decreased FOXO3A expression about 70% compared to negative control siRNA. (e) Surface CD14 and CD11b expression had clearly no change between FOXO3A siRNA and negative control siRNA in SKM-1 cells. (f) Western blot showed that FOXO3A siRNA could decrease FOXO3A expression in DAC-treated SKM-1 cells. (g) Surface CD14 expression was impaired by about 50% in FOXO3A siRNA compared with negative control siRNA in DAC-treated SKM-1 cells. ^∗^Two tailed *P* < 0.05.

The impact of DAC treatment on SKM-1 cell differentiation was examined by measuring the cell surface levels of both the monocyte differentiation marker CD14 and the myeloid cell differentiation marker CD11b before and after treatment. While we observed no CD14 expression on the surface of SKM-1 cells, more than half of the cells expressed CD11b on their surface (59.71% ± 3.80%). This CD11b expression remained constant throughout DAC treatment, whereas CD14 expression gradually increased on exposure to DAC, with the proportion of CD14-positive cells reaching a maximum of 37.19% ± 9.44% (*P* < 0.05) after 6 days of treatment (Figures 1(a) and 1(b)). FOXO3A expression in SKM-1 cells was very low in the absence of DAC, but increased statistical significance on days 3 and 6 following the initiation of treatment (Figure 1(c)). Expression of the inactive, phosphorylated form of FOXO3A (p-FOXO3A) was predominant in SKM-1 cells, indicating that FOXO3A exists primarily in the inactive form. Interestingly, DAC treatment significantly reduced the relative expression of the inactive p-FOXO3A form, resulting in a consequent increase in the FOXO3A/p-FOXO3A ratio (Figure 1(c)) and strongly indicating that DAC induces FOXO3A activation in SKM-1 cells.

Next, we investigated the role of FOXO3A in the observed DAC-induced differentiation of SKM-1 cells, by silencing FOXO3A expression with targeted siRNAs prior to DAC treatment. FOXO3A expression increased 1.77-fold in negative control siRNA-treated SKM-1 cells following treatment with DAC. Conversely, no significant difference in FOXO3A expression was seen following DAC treatment of cells treated with siRNAs targeting FOXO3A (*P* = 0.729); the expression of FOXO3A following DAC treatment was significantly lower in cells treated with the siRNA targeting FOXO3A than in cells that did not undergo NT-siRNA treatment (Figure 1(f)). These data confirm that FOXO3A expression was inhibited by the siRNA.

No significant differences in the surface expression of CD14 and CD11b were observed between SKM-1 cells where FOXO3A was silenced and nonsilenced controls (Figure 1(e)), suggesting that transient inhibition of FOXO3A expression does not affect the basal differentiation of SKM-1 cells. Interestingly, however, when FOXO3A siRNA-SKM-1 cells were treated with DAC, the observed increase in CD14-positive cells was approximately 50% lower than in cells carrying the negative control siRNA (Figure 1(g)), suggesting that silencing FOXO3A expression before DAC treatment impairs, but does not abolish, DAC-induced SKM-1 cell differentiation into monocytes. Thus, it appears that FOXO3A activation contributes to DAC-induced SKM-1 cell differentiation.

### 3.2. DAC Induces Cell Cycle Arrest in SKM-1 Cells

**Figure 2:**
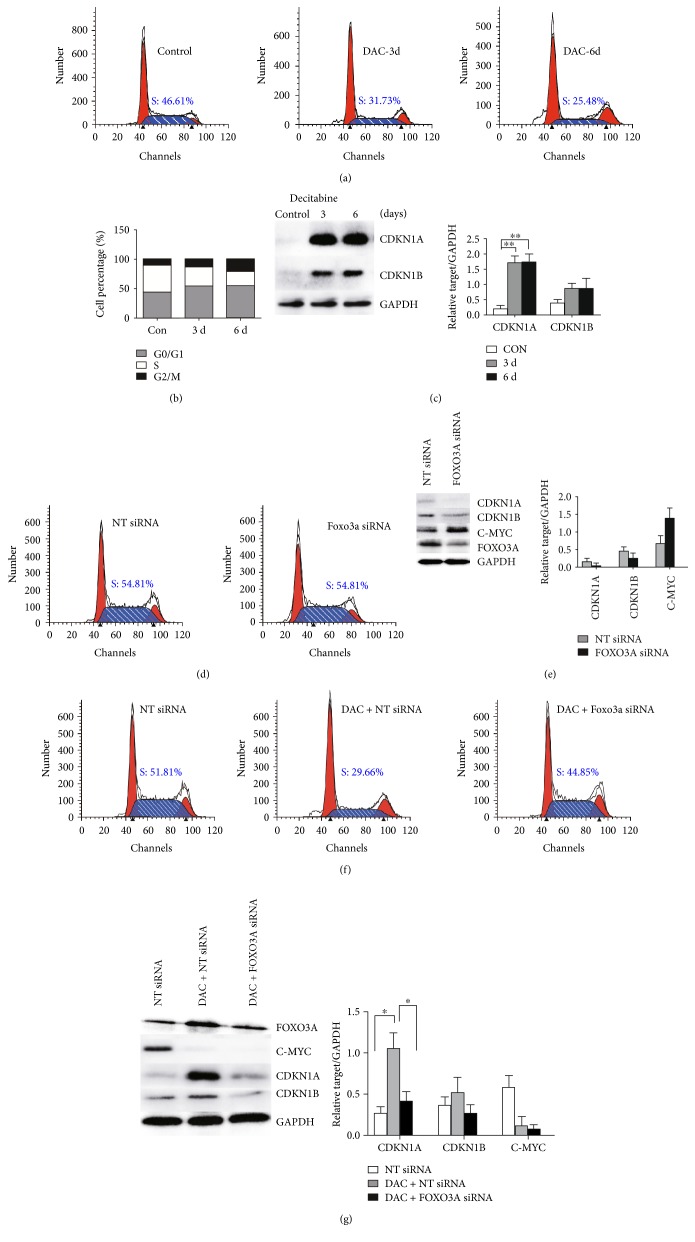
DAC induces cell cycle arrest in SKM-1 cells. (a, b) DAC treatment could decrease cells in S phase and arrest SKM-1 cells in G0/G1 and G2/M phases. (c) CDKN1A and CDKN1B levels examined in Western blot were both increased after DAC treatment in SKM-1 cells. The histogram summarizes the target/GAPDH ratio. (d) Cell cycle had clearly no change comparing FOXO3A siRNA with control siRNA. (e) Western blot showed that CDKN1A and CDKN1B levels in SKM-1 cells were decreased, but MYC was increased after FOXO3A silencing. (f) DAC-induced reduction of cells in the S phase was significantly attenuated after silencing FOXO3A. (g) DAC could not increase CDKN1A or CDKN1B protein expression in the presence of FOXO3A silencing. ^∗∗^Student's *t*-test *P* < 0.01 and ^∗^*P* < 0.05.

Having shown that DAC treatment induced differentiation in SKM-1 cells, we next investigated its impact on the cell cycle. Following DAC treatment, the proportion of cells in the S phase reduced while the proportions of cells in both G0/G1 and G2/M phases were increased, suggesting the induction of cell cycle arrest via blocks at G0/G1 and G2/M (Figures 2(a) and 2(b)). However, silencing FOXO3A before DAC treatment significantly attenuated the DAC-induced reduction of cells in the S phase (Figure 2(f)) and partly reversed the DAC-induced G0/G1 and G2/M blocks, indicating that FOXO3A is important for DAC-induced SKM-1 cell cycle arrest.

We next considered the impact of DAC treatment on gene expression. CDKN1A and CDKN1B are targeted by FOXO3A [30] and are downregulated in a variety of tumors. As shown in Figure 2(c), protein expression of both of these genes was rare in untreated SKM-1 cells, but increased after DAC treatment, especially CDKN1A (*P* < 0.01). While, compared with control siRNA, silencing FOXO3A had no significant effect on the cell cycle (Figure 2(d)), expression of the FOXO3A downstream targets CDKN1A and CDKN1B was decreased, with the effect on CDKN1A being particularly striking (Figure 2(e)). MYC is another key transcription factor that plays an important role during the G1/S phase of the cell cycle. Some studies have reported MYC to be a downstream target of FOXO3A, and, indeed, MYC activity can be inhibited by FOXO3A via the MXI1-SR*α* variant [31]. Consistent with this, MYC protein expression was promoted in cells where FOXO3A was silenced compared to in controls (Figure 2(e)). In the presence of FOXO3A silencing, DAC had no effect on either CDKN1A or CDKN1B protein expression, but MYC protein expression was downregulated (Figure 2(g)).

Overall, this suggests that DAC induces cell cycle arrest in SKM-1 cells and that the observed upregulation of CDKN1A and CDKN1B is dependent on FOXO3A, but that DAC-induced MYC downregulation is not dependent on active FOXO3A.

### 3.3. DAC Induces Apoptosis in SKM-1 Cells

**Figure 3:**
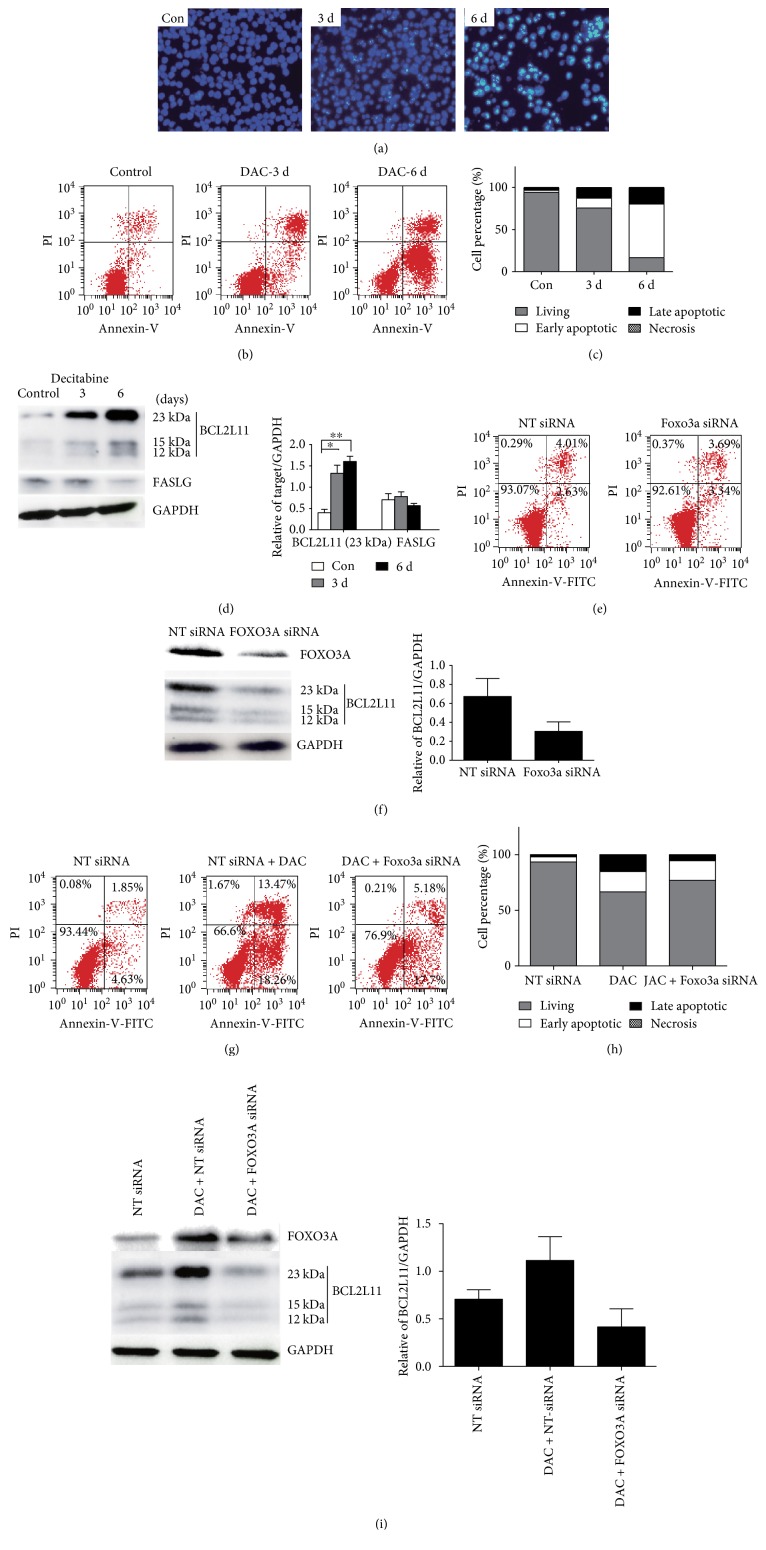
DAC induces apoptosis in SKM-1 cells. (a) Hoechst 33342 assay detected that DAC treatment could induce SKM-1 cell apoptosis. (b, c) Annexin-V-FITC/PI assay showed that apoptosis occurred in SKM-1 cells after DAC treatment. (d) BCL2L11 and FASLG proteins were examined in Western blot. BCL2L11, not FASLG, was increased after DAC treatment in SKM-1 cells. The histogram summarizes the target/GAPDH ratio. (e) Cell activity had clearly no change when compared FOXO3A siRNA with control siRNA. (f) Western blot found that BCL2L11 in SKM-1 cells were decreased after FOXO3A silencing. (g, h) FOXO3A silencing decreased the accumulation of late apoptosis of SKM-1 cells that was observed following DAC treatment. (i) BCL2L11 expression was inhibited after FOXO3A silencing and could not be induced by subsequent DAC treatment. ^∗∗^Student's *t*-test *P* < 0.01 and ^∗^*P* < 0.05.

Having shown that DAC could induce cell cycle arrest in SKM-1 cells, we investigated the effect of DAC treatment on SKM-1 apoptosis. First, DAC-treated cells were stained with Hoechst 33342 and examined using a fluorescence microscope. Apoptosis was visible from 3 days after treatment, with the apoptotic fraction reaching approximately two-thirds of the whole population by day 6 (Figure 3(a)). This trend was confirmed using Annexin-V-FITC/PI double labeling, which also showed a progressive increase in apoptosis on days 3 and 6, with the rate of increase in early apoptotic cells being most notable (Figures 3(b) and 3(c)).

Next, the expression of apoptosis-related FOXO3A downstream target proteins was examined. Detectable expression of the apoptosis-associated proteins BCL2L11 and FASLG was observed in untreated SKM-1 cells (Figure 3(d)). While DAC treatment had no significant effect on FASLG expression, BCL2L11 protein expression increased significantly after treatment, with the strongest expression seen on day 6. These results suggest that DAC induces apoptosis in SKM-1 cells primarily through the mitochondrial apoptosis pathway and that death receptor-mediated apoptosis, which features FASLG, is less important in these cells.

Annexin-V-FITC/PI double labeling indicated that silencing FOXO3A did not significantly affect apoptosis in SKM-1 cells (Figure 3(e)), although Western blot analysis did suggest that expression of the proapoptotic molecule BCL2L11 was significantly decreased after FOXO3A silencing (Figure 3(f)). Interestingly, FOXO3A silencing inhibited the accumulation of SKM-1 cells in the later stages of apoptosis that was observed following DAC treatment, but not the corresponding accumulation of cells in early apoptosis (Figures 3(d) and 3(e)), suggesting that FOXO3A might be required for the later stages, but not the early stages, of DAC-induced apoptosis. The reduction in BCL2L11 expression observed following FOXO3A silencing was not reversed by subsequent DAC treatment, suggesting a role in apoptosis that is downstream of FOXO3A activation (Figure 3(g)). As our data suggest that FOXO3A is involved only in the later stages of DAC-induced apoptosis, it is likely that BCL2L11 also plays a role at this stage.

### 3.4. FOXO3A Contributes to DAC-Mediated Autophagy in SKM-1 Cells

**Figure 4:**
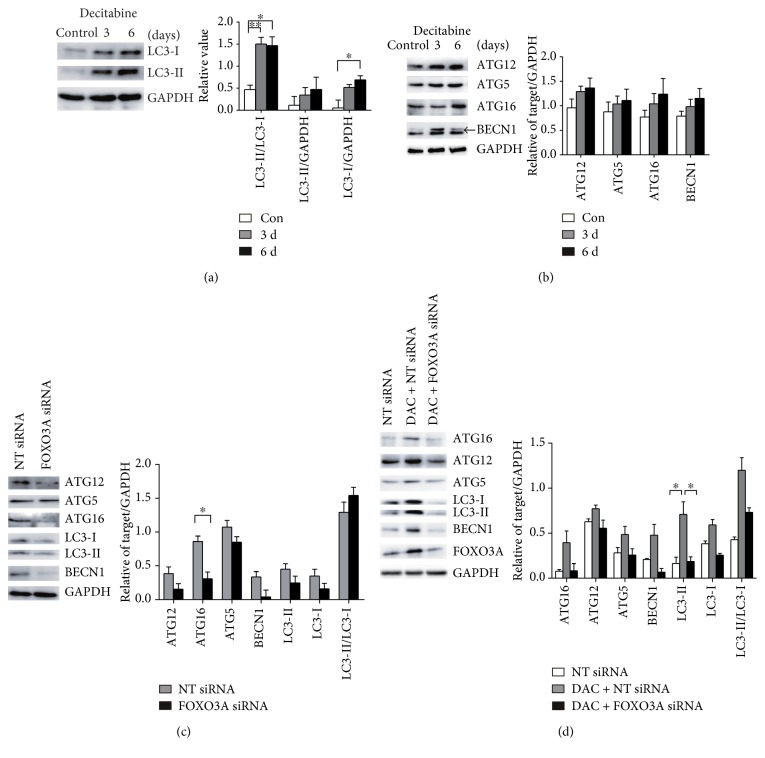
FOXO3A contributes to DAC-mediated autophagy in SKM-1 cells. (a) LC3-I and LC3-II were both increased after DAC treatment as well as the LC3-II/LC3-I ratio. (b) BECN1 and ATG5-ATG12-ATG16 complex proteins were both upregulated following DAC treatment. (c) LC3-I, LC3-II BECN1, and ATG5-ATG12-ATG16 complex proteins were decreased after FOXO3A silencing. (d) In DAC-treated SKM-1 cells, LC3-I, LC3-II BECN1, and ATG5-ATG12-ATG16 complex proteins were decreased after FOXO3A silencing. ^∗∗^Student's *t*-test *P* < 0.01 and ^∗^*P* < 0.05.

The conversion of the nonlipidated LC3 form LC3-I to the lipidated LC3-II form is a common marker of autophagic activity [32]. Western blot analysis showed that LC3-I expression was slightly higher than LC3-II expression in SKM-1 cells and that while DAC treatment induced expression of both LC3-I and LC3-II, the induction of LC3-II was more prominent, giving the increased LC3-II/LC3-I ratio that is indicative of autophagy induction (Figure 4(a)). Furthermore, the autophagy initiation protein BECN1 and the ATG5-ATG12-ATG16 complex proteins, which are associated with nucleation and elongation of the autophagosome, were both upregulated following DAC treatment, confirming the DAC-mediated induction of autophagy in SKM-1 cells (Figure 4(b)).

Following FOXO3A silencing, the expression of both LC3-I and LC3-II was reduced, but the LC3-II/LC3-I ratio was preserved. Treating the knockdown cells with DAC could not rescue expression of LC3-I but could rescue LC3-II expression to an extent; expression was restored to baseline levels but was still significantly lower than was seen in nonsilenced cells treated with DAC (Figure 4(d)). While the LC3-II/LC3-I ratio in FOXO3A-silenced cells treated with DAC was higher than in untreated silenced cells, indicating the partial induction of autophagy, this ratio remained significantly lower than in DAC-treated cells.

Further to the LC3 results, FOXO3A silencing was associated with the downregulation of ATG12, ATG16, ATG5, and BECN1 protein expression (Figure 4(c)). In contrast, DAC treatment of nonsilenced SKM-1 cells upregulated expression of these autophagy-related proteins, but this upregulation was attenuated on FOXO3A silencing resulting in lower protein expression levels. Overall, these data indicate that FOXO3A contributes to DAC-induced autophagy in SKM-1 cells via the induction of the autophagy-related genes ATG12, ATG16, ATG5, and BECN1, as silencing FOXO3A leads to a reduction in downstream effector protein expression and a partial attenuation of autophagy.

## 4. Discussion

MDS is a highly heterogeneous myeloid malignant disease that is characterized by ineffective hematopoiesis and cytopenia and that can occasionally progress into acute myeloid leukemia. While the exact mechanisms underpinning this transformation are unknown, the accumulation of genetic or epigenetic abnormalities is likely to play a role. In particular, the epigenetic silencing of tumor suppressor genes could confer a growth advantage and accelerate clonal evolution in abnormal MDS cells. In this study, we have demonstrated that FOXO3A, a potential tumor suppressor gene, is hyperphosphorylated and thus inactivated in SKM-1 cells. However, DAC treatment activated FOXO3A by both increasing expression and reducing phosphorylation, leading to the upregulation of the downstream effectors CDKN1A, CDKN1B, BCL2L11, BECN1, ATG5, ATG12, and ATG16. Consequently, DAC-induced differentiation of SKM-1 cells into monocytes, SKM-1 cell cycle arrest, apoptosis, and autophagy were observed.

We found that DAC greatly increased the expression of CDKN1A from a trace basal level in SKM-1 cells. While the CDKN1A promoter region is reportedly surrounded by CpG islands, a study of leukemic cells detected no methylation of these CpG motifs [4], suggesting that gene silencing caused by hypermethylation is not responsible for CDKN1A inactivation in leukemia cells and that other mechanisms may be involved in DAC-induced CDKN1A expression. In the ML-1 and BV-173 leukemia cell lines and the HCT116 colon cancer cell line, both of which contain wild-type TP53; DAC-induced apoptosis occurs in parallel with a TP53-dependent, but DNMT1-independent, upregulation of CDKN1A. This effect is not seen in the TP53-null HL-60 cell line, leading to the suggestion that DAC-induced CDKN1A upregulation is dependent on a DNA damage/ATM/TP53 axis [4]. As the SKM-1 cell line used in this study carries an inactive mutant form of TP53 [33], it is likely that DAC induces CDKN1A expression via other mechanisms. We observed that the expression and activity of FOXO3A, a gene that is involved in the regulation of the G0/G1 phase of the cell cycle, increased significantly after DAC treatment. We therefore speculate that the upregulation of CDKN1A, a FOXO3A target gene, is associated with the DAC-mediated activation of FOXO3A in SKM-1 cells; this theory is supported by our FOXO3A silencing data.

The protooncogenes MYC and CDKN1B play a crucial role in the control of cell cycle progression, and the induction of CDKN1B transcription by FOXO3A leads to cell cycle arrest and apoptosis. Both FOXO3A and MYC interact functionally with the forkhead binding element in the CDKN1B proximal promoter [34], meaning that MYC may inhibit the activation of CDKN1B by FOXO3A in tumor cells, potentially leading to the uncontrolled proliferation and invasiveness of a variety of tumors. MYC expression increased markedly when FOXO3A was silenced in SKM-1 cells, which could indicate that MYC is a downstream target of FOXO3A and that the tumor-suppressive properties of FOXO3A may be related to MYC inhibition. Furthermore, there is considerable overlap in the genes regulated by both FOXO3A and MYC, both in those related to growth promotion such as *CCND2*, *CDK4*, and *CCNE2* and in those related to growth inhibition such as *BBC3*, *CDKN1B*, and *GADD45A* [35, 36], suggesting that the expression of such genes is regulated by both FOXO3A and MYC in an antagonistic manner. We found that FOXO3A silencing did not affect the MYC downregulation induced by DAC, which could explain why FOXO3A silencing Foxo3a could only partially reverse DAC-induced apoptosis and cell cycle arrest in SKM-1 cells.

BCL2L11 (also known as Bim) is a proapoptotic factor containing a BH3 domain that can bind to and neutralize the antiapoptotic protein BCL2. Activated FOXO3A regulates the transcription of the *BCL2L11* gene by translocating to the nucleus, binding to the *BCL2L11* promoter, and inducing expression [37, 38]. High doses of 5-azacitidine (5-AZA; 2 *μ*M) can reportedly activate FOXO3A and thus upregulate *BCL2L11* expression and trigger apoptosis in AML cells [39]. We also observed the induction of BCL2L11 expression using low doses of DAC, although this treatment had no significant effect on FASLG expression. It is therefore likely that the apoptosis induced by low doses of DAC was mediated primarily through the mitochondrial pathway, with the death receptor pathway, which involves FASLG, playing no role in SKM-1 cells.

Autophagy, a vital mechanism for maintaining energy balance and metabolic homeostasis in cells, has a dual role in cellular biology that it can promote either cell survival or cell death. The effect of demethylation agents on tumor cell autophagy has been investigated previously, and azacitidine (AZA) treatment was found to induce apoptosis and autophagy in SKM-1 cells [40], with similar observations reported in CML cell lines [41]. In the latter study, autophagy occurred first and it was followed by apoptosis [41]. These studies, together with our results, show that demethylating agents activate cellular stress responses, eventually leading to autophagy and apoptosis. Previously, it was reported that cell death in BCR-ABL-positive CML cells was significantly increased following either treatment with an autophagy inhibitor or the silencing of the autophagy genes ATG5 and ATG7. Furthermore, the simultaneous inhibition of the Hedgehog signaling pathway and autophagy significantly reduced the activity of, and induced apoptosis in, BCR-ABL-positive CML cells, irrespective of whether they were sensitive or resistant to imatinib [42, 43]. Interestingly, silencing LC3 in MDS cell lines significantly increased AZA-induced cell death, indicating that AZA-induced autophagy may be a protective, rather than cytotoxic, mechanism in cells [44] and that the AZA-induced autophagy observed by Cluzeau et al. [40] may not be contributing to cell death. It is possible that the autophagy occurring with other traditional chemotherapy drugs, as with AZA, may constitute a compensatory mechanism to protect cells from drug-induced damage and apoptosis. It is therefore not possible to determine whether the autophagy that is observed in SKM-1 cells in response to DAC treatment is a drug-induced protective stress response or a cytotoxic response.

## 5. Conclusion

This study showed that silencing FOXO3A expression impaired DAC-induced cellular differentiation, cell cycle arrest, and apoptosis, potentially because of the observed downregulation of CDKN1B, CDKN1A, and BCL2L11. Interestingly, DAC-induced MYC downregulation was not reversed by FOXO3A silencing, which could explain the partial reversal of DAC-induced cell cycle arrest and apoptosis that is observed when FOXO3A expression is lost. The upregulation of the autophagy-related proteins BECN1, ATG5, ATG12, and ATG16 in SKM-1 cells following DAC treatment, as well as the consequent increase in autophagy, was found to be related to the DAC-induced upregulation and activation of FOXO3A. Collectively, these results suggest that DAC-reactivation of FOXO3A has potential in MDS therapeutics.

## Supplementary Material

Supplementary Table 1: Primary antibodies and its sources.

## References

[1] Wang M., Zhang X., Zhao H., Wang Q., Pan Y. (2009). FoxO gene family evolution in vertebrates. *BMC Evolutionary Biology*.

[2] Korashy H. M., Belali O. M., Ansar M. A., Alharbi N. O. (2016). FoxO3a is essential for the antiproliferative and apoptogenic effects of sunitinib in MDA-MB231 cell line. *Anticancer Research*.

[3] Qian K., Wang G., Cao R. (2016). Capsaicin suppresses cell proliferation, induces cell cycle arrest and ROS production in bladder cancer cells through FOXO3a-mediated pathways. *Molecules*.

[4] Qiang W., Sui F., Ma J. (2017). Proteasome inhibitor MG132 induces thyroid cancer cell apoptosis by modulating the activity of transcription factor FOXO3a. *Endocrine*.

[5] Nguyen L. T., Lee Y. H., Sharma A. R. (2017). Quercetin induces apoptosis and cell cycle arrest in triple-negative breast cancer cells through modulation of Foxo3a activity. *The Korean Journal of Physiology & Pharmacology*.

[6] Nestal de Moraes G., Bella L., Zona S., Burton M. J., Lam E. W. (2016). Insights into a critical role of the FOXO3a-FOXM1 axis in DNA damage response and genotoxic drug resistance. *Current Drug Targets*.

[7] Kim H. J., Lee S. Y., Kim C. Y., Kim Y. H., Ju W., Kim S. C. (2017). Subcellular localization of FOXO3a as a potential biomarker of response to combined treatment with inhibitors of PI3K and autophagy in PIK3CA-mutant cancer cells. *Oncotarget*.

[8] Sun L., Zhao M., Wang Y. (2017). Neuroprotective effects of miR-27a against traumatic brain injury via suppressing FoxO3a-mediated neuronal autophagy. *Biochemical and Biophysical Research Communications*.

[9] Chen Y., Lv L., Pi H. (2016). Dihydromyricetin protects against liver ischemia/reperfusion induced apoptosis via activation of FOXO3a-mediated autophagy. *Oncotarget*.

[10] Van Der Heide L. P., Hoekman M. F., Smidt M. P. (2004). The ins and outs of FoxO shuttling: mechanisms of FoxO translocation and transcriptional regulation. *Biochemical Journal*.

[11] Brownawell A. M., Kops G. J., Macara I. G., Burgering B. M. (2001). Inhibition of nuclear import by protein kinase B (Akt) regulates the subcellular distribution and activity of the forkhead transcription factor AFX. *Molecular and Cellular Biology*.

[12] Biggs W. H., Cavenee W. K., Arden K. C. (2001). Identification and characterization of members of the FKHR (FOX O) subclass of winged-helix transcription factors in the mouse. *Mammalian Genome*.

[13] Marfe G., Tafani M., Fiorito F., Pagnini U., Iovane G., De Martino L. (2011). Involvement of FOXO transcription factors, TRAIL-FasL/Fas, and sirtuin proteins family in canine coronavirus type II-induced apoptosis. *PLoS One*.

[14] Wattel E., Preudhomme C., Hecquet B. (1994). p53 mutations are associated with resistance to chemotherapy and short survival in hematologic malignancies. *Blood*.

[15] Fang Y., Yang Y., Hua C. (2017). Rictor plays a pivotal role in maintaining quiescence as well as stemness of leukemia stem cells in MLL-driven leukemia. *Leukemia*.

[16] Hu C., Ni Z., Li B. S. (2015). hTERT promotes the invasion of gastric cancer cells by enhancing FOXO3a ubiquitination and subsequent ITGB1 upregulation. *Gut*.

[17] Chapuis N., Park S., Leotoing L. (2010). I*κ*B kinase overcomes PI3K/Akt and ERK/MAPK to control FOXO3a activity in acute myeloid leukemia. *Blood*.

[18] Stankiewicz M. J., Crispino J. D. (2013). AKT collaborates with ERG and Gata1s to dysregulate megakaryopoiesis and promote AMKL. *Leukemia*.

[19] Tubi L. Q., Nunes S. C., Brancalion A. (2016). Protein kinase CK2 regulates AKT, NF-κB and STAT3 activation, stem cell viability and proliferation in acute myeloid leukemia. *Leukemia*.

[20] Ranganathan P., Yu X., Santhanam R. (2015). Decitabine priming enhances the antileukemic effects of exportin 1 (XPO1) selective inhibitor selinexor in acute myeloid leukemia. *Blood*.

[21] Schmelz K., Wagner M., Dorken B., Tamm I. (2005). 5-Aza-2′-deoxycytidine induces p21WAF expression by demethylation of p73 leading to p53-independent apoptosis in myeloid leukemia. *International Journal of Cancer*.

[22] Covey J. M., D'Incalci M., Tilchen E. J., Zaharko D. S., Kohn K. W. (1986). Differences in DNA damage produced by incorporation of 5-aza-2′-deoxycytidine or 5,6-dihydro-5-azacytidine into DNA of mammalian cells. *Cancer Research*.

[23] Schermelleh L., Haemmer A., Spada F. (2007). Dynamics of Dnmt1 interaction with the replication machinery and its role in postreplicative maintenance of DNA methylation. *Nucleic Acids Research*.

[24] Hollenbach P. W., Nguyen A. N., Brady H. (2010). A comparison of azacitidine and decitabine activities in acute myeloid leukemia cell lines. *PLoS One*.

[25] Alcazar O., Achberger S., Aldrich W. (2012). Epigenetic regulation by decitabine of melanoma differentiation in vitro and in vivo. *International Journal of Cancer*.

[26] Marcucci G., Silverman L., Eller M., Lintz L., Beach C. L. (2005). Bioavailability of azacitidine subcutaneous versus intravenous in patients with the myelodysplastic syndromes. *Journal of Clinical Pharmacology*.

[27] Cashen A. F., Shah A. K., Todt L., Fisher N., DiPersio J. (2008). Pharmacokinetics of decitabine administered as a 3-h infusion to patients with acute myeloid leukemia (AML) or myelodysplastic syndrome (MDS). *Cancer Chemotherapy and Pharmacology*.

[28] Blum W., Klisovic R. B., Hackanson B. (2007). Phase I study of decitabine alone or in combination with valproic acid in acute myeloid leukemia. *Journal of Clinical Oncology*.

[29] Ng K. P., Ebrahem Q., Negrotto S. (2011). p53 independent epigenetic-differentiation treatment in xenotransplant models of acute myeloid leukemia. *Leukemia*.

[30] McClelland Descalzo D. L., Satoorian T. S., Walker L. M., Sparks N. R., Pulyanina P. Y., Zur Nieden N. I. (2016). Glucose-induced oxidative stress reduces proliferation in embryonic stem cells via FOXO3A/*β*-catenin-dependent transcription of *p21*^*cip1*^. *Stem Cell Reports*.

[31] Gan B., Lim C., Chu G. (2010). FoxOs enforce a progression checkpoint to constrain mTORC1-activated renal tumorigenesis. *Cancer Cell*.

[32] Shin H. J., Kim H., Oh S. (2016). AMPK-SKP2-CARM1 signalling cascade in transcriptional regulation of autophagy. *Nature*.

[33] Cluzeau T., Dubois A., Jacquel A. (2014). Phenotypic and genotypic characterization of azacitidine-sensitive and resistant SKM1 myeloid cell lines. *Oncotarget*.

[34] Chandramohan V., Mineva N. D., Burke B. (2008). c-Myc represses FOXO3a-mediated transcription of the gene encoding the p27Kip1 cyclin dependent kinase inhibitor. *Journal of Cellular Biochemistry*.

[35] Bouchard C., Marquardt J., Brás A., Medema R. H., Eilers M. (2004). Myc-induced proliferation and transformation require Akt-mediated phosphorylation of FoxO proteins. *EMBO Journal*.

[36] Amente S., Zhang J., Lavadera M. L., Lania L., Avvedimento E. V., Majello B. (2011). Myc and PI3K/AKT signaling cooperatively repress FOXO3a-dependent PUMA and GADD45a gene expression. *Nucleic Acids Research*.

[37] You H., Pellegrini M., Tsuchihara K. (2006). FOXO3a-dependent regulation of Puma in response to cytokine/growth factor withdrawal. *The Journal of Experimental Medicine*.

[38] Dijkers P. F., Medema R. H., Lammers J. W., Koenderman L., Coffer P. J. (2000). Expression of the pro-apoptotic Bcl-2 family member Bim is regulated by the forkhead transcription factor FKHR-L1. *Current Biology*.

[39] Thepot S., Lainey E., Cluzeau T. (2011). Hypomethylating agents reactivate FOXO3A in acute myeloid leukemia. *Cell Cycle*.

[40] Cluzeau T., Robert G., Puissant A. (2011). Azacitidine-resistant SKM1 myeloid cells are defective for AZA-induced mitochondrial apoptosis and autophagy. *Cell Cycle*.

[41] Schnekenburger M., Grandjenette C., Ghelfi J. (2011). Sustained exposure to the DNA demethylating agent, 2′-deoxy-5-azacytidine, leads to apoptotic cell death in chronic myeloid leukemia by promoting differentiation, senescence, and autophagy. *Biochemical Pharmacology*.

[42] Zeng X., Zhao H., Li Y. (2015). Targeting Hedgehog signaling pathway and autophagy overcomes drug resistance of BCR-ABL-positive chronic myeloid leukemia. *Autophagy*.

[43] Hart L. S., Cunningham J. T., Datta T. (2012). ER stress–mediated autophagy promotes Myc-dependent transformation and tumor growth. *The Journal of Clinical Investigation*.

[44] Kroemer G., Marino G., Levine B. (2010). Autophagy and the integrated stress response. *Molecular Cell*.

